# *N*-Benzyl-4-methyl-6-phenyl­pyrimidin-2-amine

**DOI:** 10.1107/S1600536811041365

**Published:** 2011-10-12

**Authors:** Hoong-Kun Fun, Madhukar Hemamalini, Anita Hazra, Shyamaprosad Goswami

**Affiliations:** a X-ray Crystallography Unit, School of Physics, Universiti Sains Malaysia, 11800 USM, Penang, Malaysia; b Department of Chemistry, Bengal Engineering and Science University, Shibpur, Howrah 711 103, India

## Abstract

In the title compound, C_18_H_17_N_3_, the dihedral angles between the central pyrimidine ring and its directly-bonded and N-bonded pendant phenyl rings are 25.48 (6) and 80.33 (6)°, respectively. The dihedral angle between the phenyl rings is 79.66 (6)°. In the crystal, inversion dimers linked by pairs of N—H⋯N hydrogen bonds generate *R*_2_^2^(8) loops. The crystal structure also features weak π–π [centroid–centroid separation = 3.6720 (7) Å] and C—H⋯π inter­actions.

## Related literature

For background to pyrimidine derivatives, see: Katrizky (1982 ▶); Brown & Lyall (1964 ▶). For a related structure, see: Goswami *et al.* (2009 ▶). For graph-set notation, see: Bernstein *et al.* (1995 ▶). For the stability of the temperature controller used in the data collection, see: Cosier & Glazer (1986 ▶).
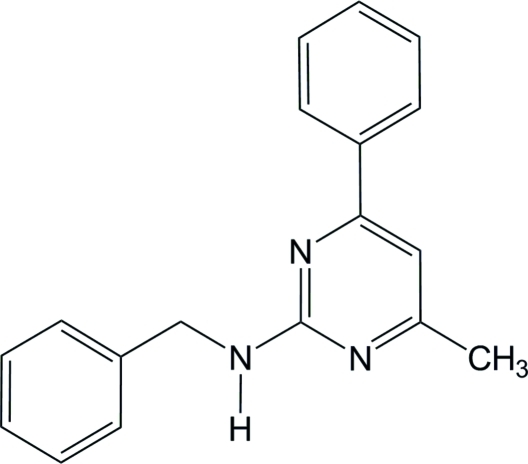


## Experimental

### 

#### Crystal data

C_18_H_17_N_3_
*M* *_r_* = 275.35
Triclinic, 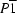

*a* = 8.2974 (1) Å
*b* = 9.9316 (2) Å
*c* = 10.7251 (2) Å
α = 115.797 (1)°
β = 93.019 (1)°
γ = 111.565 (1)°
*V* = 715.78 (2) Å^3^
*Z* = 2
Mo *K*α radiation
μ = 0.08 mm^−1^
*T* = 100 K
0.31 × 0.23 × 0.20 mm

#### Data collection

Bruker SMART APEXII CCD diffractometer
Absorption correction: multi-scan (*SADABS*; Bruker, 2009 ▶) *T*_min_ = 0.976, *T*_max_ = 0.985
14761 measured reflections
3272 independent reflections
2882 reflections with *I* > 2σ(*I*)
*R*_int_ = 0.028

#### Refinement

*R*[*F* ^2^ > 2σ(*F* ^2^)] = 0.038
*wR*(*F* ^2^) = 0.103
*S* = 1.08
3272 reflections
258 parameters
All H-atom parameters refined
Δρ_max_ = 0.30 e Å^−3^
Δρ_min_ = −0.23 e Å^−3^

### 

Data collection: *APEX2* (Bruker, 2009 ▶); cell refinement: *SAINT* (Bruker, 2009 ▶); data reduction: *SAINT*; program(s) used to solve structure: *SHELXTL* (Sheldrick, 2008 ▶); program(s) used to refine structure: *SHELXTL*; molecular graphics: *SHELXTL*; software used to prepare material for publication: *SHELXTL* and *PLATON* (Spek, 2009 ▶).

## Supplementary Material

Crystal structure: contains datablock(s) global, I. DOI: 10.1107/S1600536811041365/hb6441sup1.cif

Structure factors: contains datablock(s) I. DOI: 10.1107/S1600536811041365/hb6441Isup2.hkl

Supplementary material file. DOI: 10.1107/S1600536811041365/hb6441Isup3.cml

Additional supplementary materials:  crystallographic information; 3D view; checkCIF report

## Figures and Tables

**Table 1:** Hydrogen-bond geometry (Å, °) *Cg*1 is the centroid of the N1,N2/C7–C10 ring. *Cg*3 is the centroid of the C12–C17 ring.

*D*—H⋯*A*	*D*—H	H⋯*A*	*D*⋯*A*	*D*—H⋯*A*
N3—H1*N*3⋯N1^i^	0.909 (17)	2.147 (17)	3.0539 (14)	175.7 (14)
C5—H5*A*⋯*Cg*1^ii^	0.995 (14)	2.883 (15)	3.3595 (14)	110.3 (10)
C18—H18*A*⋯*Cg*3^iii^	0.960 (16)	2.846 (19)	3.7977 (16)	171.8 (13)

Symmetry codes: (i) 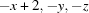; (ii) 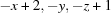; (iii) 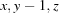.

**Fig. 1.**
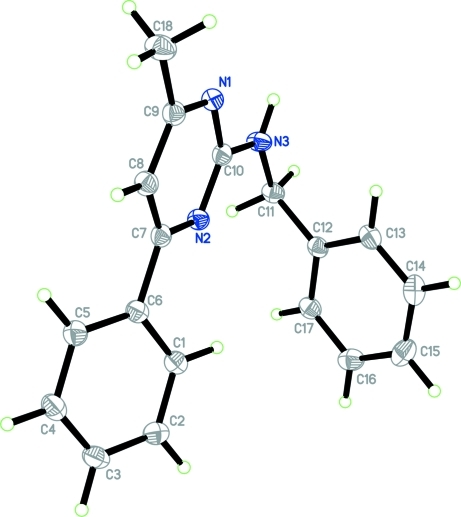
The asymmetric unit of (I), showing 50% probability displacement ellipsoids.

**Fig. 2.**
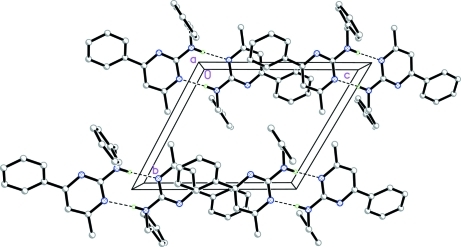
The crystal packing of the title compound (I).

## supplementary crystallographic information

### Comment

Substituted pyrimidine derivatives are important components of various bioactive molecules (Katrizky, 1982; Brown & Lyall, 1964). We have synthesised benzyl-(4-methyl-6-phenyl-pyrimidin-2-yl)-amine by solid-phase microwave irradiation (Goswami *et al.*, 2009). Herein, we wish to report the crystal structure of the title compound, (I), (Fig. 1).

The central pyrimidine (N1,N2/C7–C10) ring makes dihedral angles of 25.48 (6) and 80.33 (6)° with the terminal phenyl (C1–C6/C12–C17) rings. The corresponding angle between the two terminal phenyl (C1–C6/C10–C15) rings is 79.66 (6)°.

In the crystal (Fig. 2), centrosymmetrically-related molecules are linked into dimers *via* pairs of N—H···N hydrogen bonds (Table 1), generating *R*_2_^2^(8) ring motifs. (Bernstein *et al.*, 1995).   The crystal structure is further stabilized by π–π interactions between the benzene (Cg2; C1–C6) rings [Cg2···Cg2 = 3.6720 (7) Å; 1-x, -y, 1-z] and C—H···π interaction involving the centroids of the N1,N2/C7–C10 (Cg1) and C12–C17 (Cg3) rings.

### Experimental

A mixture of *S*-methylisothiourea sulphate (556 mg, 2 mmol), potassium carbonate (345 mg, 2.5 mmol) and benzylamine ((428 mg, 4 mmol) was irradiated at 450 Watt for 18 minutes in a microwave oven. The solid mass was washed with chloroform to remove the unreacted benzylamine and then dried. The solid residue was then mixed with benzoyl acetone (648 mg, 4 mmol) and again irradiated at 300 Watt for 5 minutes. Water was added to it and the contents were extracted with chloroform. The crude product was then purified through column chromatography (silica gel, 100–200 mesh) using 12% ethyl acetate in petroleum ether as an eluent to afford the pure compound. Colourless blocks of (I) were grown by slow evaporation of a chloroform and methanol (3:1) solution. Mp 112–114°C.

### Refinement

All hydrogen atoms were located from a difference Fourier maps and refined freely [N–H = 0.909 (16) Å and C–H = 0.960 (16)– 1.008 (18) Å]. The highest residual electron density peak is located at 0.75 Å from C18 and the deepest hole 0.67 Å located at from C11.

### Crystal data

**Table:**

C_18_H_17_N_3_	*Z* = 2
*M**_r_* = 275.35	*F*(000) = 292
Triclinic, *P*1	*D*_x_ = 1.278 Mg m^−^^3^
Hall symbol: -P 1	Mo *K*α radiation, λ = 0.71073 Å
*a* = 8.2974 (1) Å	Cell parameters from 8187 reflections
*b* = 9.9316 (2) Å	θ = 2.4–32.6°
*c* = 10.7251 (2) Å	µ = 0.08 mm^−^^1^
α = 115.797 (1)°	*T* = 100 K
β = 93.019 (1)°	Block, colourless
γ = 111.565 (1)°	0.31 × 0.23 × 0.20 mm
*V* = 715.78 (2) Å^3^	

### Data collection

**Table:**

Bruker SMART APEXII CCD diffractometer	3272 independent reflections
Radiation source: fine-focus sealed tube	2882 reflections with *I* > 2σ(*I*)
graphite	*R*_int_ = 0.028
φ and ω scans	θ_max_ = 27.5°, θ_min_ = 2.2°
Absorption correction: multi-scan (*SADABS*; Bruker, 2009)	*h* = −10→10
*T*_min_ = 0.976, *T*_max_ = 0.985	*k* = −12→12
14761 measured reflections	*l* = −13→13

### Refinement

**Table:**

Refinement on *F*^2^	Primary atom site location: structure-invariant direct methods
Least-squares matrix: full	Secondary atom site location: difference Fourier map
*R*[*F*^2^ > 2σ(*F*^2^)] = 0.038	Hydrogen site location: inferred from neighbouring sites
*wR*(*F*^2^) = 0.103	All H-atom parameters refined
*S* = 1.08	*w* = 1/[σ^2^(*F*_o_^2^) + (0.0489*P*)^2^ + 0.2057*P*] where *P* = (*F*_o_^2^ + 2*F*_c_^2^)/3
3272 reflections	(Δ/σ)_max_ < 0.001
258 parameters	Δρ_max_ = 0.30 e Å^−^^3^
0 restraints	Δρ_min_ = −0.23 e Å^−^^3^

### Special details

**Table:**

Experimental. The crystal was placed in the cold stream of an Oxford Cryosystems Cobra open-flow nitrogen cryostat (Cosier & Glazer, 1986) operating at 100.0 (1) K.
Geometry. All s.u.'s (except the s.u. in the dihedral angle between two l.s. planes) are estimated using the full covariance matrix. The cell s.u.'s are taken into account individually in the estimation of s.u.'s in distances, angles and torsion angles; correlations between s.u.'s in cell parameters are only used when they are defined by crystal symmetry. An approximate (isotropic) treatment of cell s.u.'s is used for estimating s.u.'s involving l.s. planes.
Refinement. Refinement of F^2^ against ALL reflections. The weighted R-factor wR and goodness of fit S are based on F^2^, conventional R-factors R are based on F, with F set to zero for negative F^2^. The threshold expression of F^2^ > 2σ(F^2^) is used only for calculating R-factors(gt) etc. and is not relevant to the choice of reflections for refinement. R-factors based on F^2^ are statistically about twice as large as those based on F, and R- factors based on ALL data will be even larger.

### Fractional atomic coordinates and isotropic or equivalent isotropic displacement parameters (Å^2^)

**Table:**

	*x*	*y*	*z*	*U*_iso_*/*U*_eq_	
N1	0.90066 (12)	−0.08503 (11)	0.12219 (9)	0.0188 (2)	
N2	0.87677 (11)	0.12752 (11)	0.33305 (9)	0.0183 (2)	
N3	0.97171 (13)	0.17646 (11)	0.15239 (10)	0.0212 (2)	
C1	0.71112 (14)	0.21029 (13)	0.56032 (11)	0.0198 (2)	
C2	0.65631 (15)	0.26533 (14)	0.68605 (12)	0.0223 (2)	
C3	0.65182 (15)	0.19273 (14)	0.77178 (12)	0.0238 (2)	
C4	0.70214 (15)	0.06358 (15)	0.73105 (12)	0.0242 (2)	
C5	0.75556 (15)	0.00702 (14)	0.60459 (12)	0.0218 (2)	
C6	0.76074 (13)	0.07970 (13)	0.51815 (11)	0.0184 (2)	
C7	0.81314 (14)	0.01877 (13)	0.38081 (11)	0.0180 (2)	
C8	0.79212 (14)	−0.14363 (14)	0.30300 (12)	0.0204 (2)	
C9	0.83871 (14)	−0.19054 (13)	0.17325 (11)	0.0199 (2)	
C10	0.91539 (13)	0.07005 (13)	0.20488 (11)	0.0182 (2)	
C11	0.98525 (15)	0.34359 (13)	0.22902 (12)	0.0205 (2)	
C12	0.80780 (14)	0.35250 (13)	0.24133 (11)	0.0187 (2)	
C13	0.65081 (15)	0.23457 (14)	0.13426 (12)	0.0223 (2)	
C14	0.48991 (16)	0.24699 (15)	0.14611 (13)	0.0269 (3)	
C15	0.48407 (16)	0.37634 (16)	0.26597 (14)	0.0280 (3)	
C16	0.64009 (16)	0.49505 (14)	0.37303 (13)	0.0250 (3)	
C17	0.80155 (15)	0.48400 (13)	0.36038 (12)	0.0210 (2)	
C18	0.81962 (18)	−0.36362 (15)	0.08273 (13)	0.0273 (3)	
H1N3	1.005 (2)	0.1438 (18)	0.0687 (17)	0.033 (4)*	
H1A	0.7119 (17)	0.2595 (15)	0.4979 (14)	0.019 (3)*	
H2A	0.6173 (18)	0.3557 (17)	0.7140 (15)	0.027 (3)*	
H3A	0.6092 (19)	0.2292 (17)	0.8604 (16)	0.032 (4)*	
H4A	0.7012 (19)	0.0135 (17)	0.7921 (15)	0.029 (4)*	
H5A	0.7917 (18)	−0.0852 (17)	0.5761 (14)	0.024 (3)*	
H8A	0.7447 (18)	−0.2220 (17)	0.3358 (14)	0.026 (3)*	
H11A	1.0675 (18)	0.4045 (16)	0.3265 (15)	0.024 (3)*	
H11B	1.0416 (18)	0.4018 (16)	0.1759 (14)	0.023 (3)*	
H13A	0.6563 (17)	0.1413 (16)	0.0507 (14)	0.022 (3)*	
H14A	0.379 (2)	0.1611 (18)	0.0700 (16)	0.033 (4)*	
H15A	0.369 (2)	0.3830 (19)	0.2750 (16)	0.038 (4)*	
H16A	0.6346 (19)	0.5878 (18)	0.4579 (16)	0.032 (4)*	
H17A	0.9114 (19)	0.5707 (17)	0.4357 (15)	0.025 (3)*	
H18A	0.763 (2)	−0.4358 (19)	0.1203 (16)	0.038 (4)*	
H18B	0.749 (2)	−0.414 (2)	−0.0168 (19)	0.047 (4)*	
H18C	0.939 (2)	−0.366 (2)	0.0729 (18)	0.050 (5)*	

### Atomic displacement parameters (Å^2^)

**Table:**

	*U*^11^	*U*^22^	*U*^33^	*U*^12^	*U*^13^	*U*^23^
N1	0.0189 (4)	0.0202 (4)	0.0175 (4)	0.0093 (4)	0.0045 (3)	0.0088 (4)
N2	0.0171 (4)	0.0204 (4)	0.0173 (4)	0.0082 (4)	0.0051 (3)	0.0090 (4)
N3	0.0274 (5)	0.0207 (5)	0.0203 (5)	0.0127 (4)	0.0119 (4)	0.0114 (4)
C1	0.0185 (5)	0.0198 (5)	0.0189 (5)	0.0062 (4)	0.0046 (4)	0.0094 (4)
C2	0.0205 (5)	0.0216 (5)	0.0221 (5)	0.0086 (4)	0.0064 (4)	0.0087 (4)
C3	0.0215 (5)	0.0275 (6)	0.0172 (5)	0.0086 (5)	0.0063 (4)	0.0082 (4)
C4	0.0252 (5)	0.0286 (6)	0.0196 (5)	0.0099 (5)	0.0060 (4)	0.0138 (5)
C5	0.0210 (5)	0.0231 (5)	0.0209 (5)	0.0096 (4)	0.0046 (4)	0.0107 (4)
C6	0.0147 (5)	0.0194 (5)	0.0167 (5)	0.0047 (4)	0.0029 (4)	0.0076 (4)
C7	0.0145 (4)	0.0211 (5)	0.0180 (5)	0.0072 (4)	0.0028 (4)	0.0098 (4)
C8	0.0211 (5)	0.0216 (5)	0.0210 (5)	0.0092 (4)	0.0065 (4)	0.0125 (4)
C9	0.0193 (5)	0.0200 (5)	0.0196 (5)	0.0085 (4)	0.0035 (4)	0.0092 (4)
C10	0.0148 (5)	0.0216 (5)	0.0183 (5)	0.0082 (4)	0.0035 (4)	0.0097 (4)
C11	0.0227 (5)	0.0192 (5)	0.0202 (5)	0.0086 (4)	0.0074 (4)	0.0103 (4)
C12	0.0227 (5)	0.0194 (5)	0.0177 (5)	0.0092 (4)	0.0065 (4)	0.0118 (4)
C13	0.0272 (6)	0.0205 (5)	0.0182 (5)	0.0096 (4)	0.0037 (4)	0.0096 (4)
C14	0.0237 (6)	0.0258 (6)	0.0297 (6)	0.0070 (5)	−0.0002 (5)	0.0162 (5)
C15	0.0239 (6)	0.0317 (6)	0.0395 (7)	0.0152 (5)	0.0113 (5)	0.0237 (6)
C16	0.0310 (6)	0.0232 (6)	0.0277 (6)	0.0149 (5)	0.0133 (5)	0.0148 (5)
C17	0.0245 (5)	0.0191 (5)	0.0191 (5)	0.0081 (4)	0.0061 (4)	0.0102 (4)
C18	0.0380 (7)	0.0222 (6)	0.0237 (6)	0.0144 (5)	0.0117 (5)	0.0113 (5)

### Geometric parameters (Å, °)

**Table:**

N1—C9	1.3399 (14)	C8—C9	1.3884 (15)
N1—C10	1.3546 (13)	C8—H8A	0.955 (14)
N2—C7	1.3411 (14)	C9—C18	1.5012 (15)
N2—C10	1.3476 (14)	C11—C12	1.5163 (15)
N3—C10	1.3545 (14)	C11—H11A	0.998 (14)
N3—C11	1.4522 (13)	C11—H11B	0.996 (14)
N3—H1N3	0.909 (16)	C12—C13	1.3920 (15)
C1—C2	1.3892 (15)	C12—C17	1.3943 (15)
C1—C6	1.3992 (15)	C13—C14	1.3925 (17)
C1—H1A	0.985 (13)	C13—H13A	0.988 (13)
C2—C3	1.3883 (17)	C14—C15	1.3858 (18)
C2—H2A	0.995 (14)	C14—H14A	0.990 (15)
C3—C4	1.3913 (17)	C15—C16	1.3884 (17)
C3—H3A	0.994 (15)	C15—H15A	0.987 (16)
C4—C5	1.3909 (16)	C16—C17	1.3926 (16)
C4—H4A	0.978 (15)	C16—H16A	0.992 (14)
C5—C6	1.3951 (16)	C17—H17A	0.982 (14)
C5—H5A	0.995 (14)	C18—H18A	0.960 (16)
C6—C7	1.4866 (15)	C18—H18B	0.993 (17)
C7—C8	1.3909 (15)	C18—H18C	1.008 (18)
			
C9—N1—C10	115.99 (9)	N2—C10—N1	126.24 (10)
C7—N2—C10	116.49 (9)	N3—C10—N1	116.72 (9)
C10—N3—C11	121.48 (9)	N3—C11—C12	114.71 (9)
C10—N3—H1N3	119.4 (9)	N3—C11—H11A	109.8 (7)
C11—N3—H1N3	119.1 (9)	C12—C11—H11A	109.6 (7)
C2—C1—C6	120.17 (10)	N3—C11—H11B	107.0 (7)
C2—C1—H1A	120.9 (7)	C12—C11—H11B	108.3 (7)
C6—C1—H1A	118.9 (7)	H11A—C11—H11B	107.2 (11)
C3—C2—C1	120.50 (11)	C13—C12—C17	118.88 (10)
C3—C2—H2A	119.6 (8)	C13—C12—C11	121.50 (9)
C1—C2—H2A	119.9 (8)	C17—C12—C11	119.60 (10)
C2—C3—C4	119.66 (10)	C12—C13—C14	120.63 (10)
C2—C3—H3A	120.9 (8)	C12—C13—H13A	118.3 (8)
C4—C3—H3A	119.4 (8)	C14—C13—H13A	121.0 (8)
C5—C4—C3	120.06 (11)	C15—C14—C13	120.17 (11)
C5—C4—H4A	120.1 (8)	C15—C14—H14A	120.0 (8)
C3—C4—H4A	119.8 (8)	C13—C14—H14A	119.8 (8)
C4—C5—C6	120.55 (10)	C14—C15—C16	119.64 (11)
C4—C5—H5A	119.7 (8)	C14—C15—H15A	119.9 (9)
C6—C5—H5A	119.7 (8)	C16—C15—H15A	120.5 (9)
C5—C6—C1	119.06 (10)	C15—C16—C17	120.23 (11)
C5—C6—C7	121.54 (10)	C15—C16—H16A	119.0 (8)
C1—C6—C7	119.38 (10)	C17—C16—H16A	120.8 (8)
N2—C7—C8	121.45 (10)	C16—C17—C12	120.44 (10)
N2—C7—C6	116.37 (9)	C16—C17—H17A	119.2 (8)
C8—C7—C6	122.15 (10)	C12—C17—H17A	120.3 (8)
C9—C8—C7	117.87 (10)	C9—C18—H18A	112.6 (9)
C9—C8—H8A	120.5 (8)	C9—C18—H18B	111.5 (10)
C7—C8—H8A	121.7 (8)	H18A—C18—H18B	108.1 (13)
N1—C9—C8	121.93 (10)	C9—C18—H18C	112.2 (10)
N1—C9—C18	116.91 (10)	H18A—C18—H18C	107.2 (13)
C8—C9—C18	121.15 (10)	H18B—C18—H18C	104.9 (13)
N2—C10—N3	117.03 (9)		
			
C6—C1—C2—C3	0.63 (16)	C7—C8—C9—C18	−179.86 (10)
C1—C2—C3—C4	−0.19 (17)	C7—N2—C10—N3	176.97 (9)
C2—C3—C4—C5	−0.40 (17)	C7—N2—C10—N1	−1.94 (15)
C3—C4—C5—C6	0.55 (17)	C11—N3—C10—N2	−1.62 (15)
C4—C5—C6—C1	−0.10 (16)	C11—N3—C10—N1	177.39 (9)
C4—C5—C6—C7	−178.51 (10)	C9—N1—C10—N2	1.20 (15)
C2—C1—C6—C5	−0.49 (16)	C9—N1—C10—N3	−177.71 (9)
C2—C1—C6—C7	177.95 (9)	C10—N3—C11—C12	−66.03 (13)
C10—N2—C7—C8	1.35 (15)	N3—C11—C12—C13	−32.47 (15)
C10—N2—C7—C6	−176.58 (9)	N3—C11—C12—C17	149.37 (10)
C5—C6—C7—N2	−156.38 (10)	C17—C12—C13—C14	−0.39 (17)
C1—C6—C7—N2	25.21 (14)	C11—C12—C13—C14	−178.56 (10)
C5—C6—C7—C8	25.70 (15)	C12—C13—C14—C15	−0.80 (18)
C1—C6—C7—C8	−152.70 (10)	C13—C14—C15—C16	1.10 (18)
N2—C7—C8—C9	−0.20 (15)	C14—C15—C16—C17	−0.22 (18)
C6—C7—C8—C9	177.61 (9)	C15—C16—C17—C12	−0.98 (17)
C10—N1—C9—C8	0.13 (15)	C13—C12—C17—C16	1.27 (16)
C10—N1—C9—C18	179.44 (9)	C11—C12—C17—C16	179.48 (10)
C7—C8—C9—N1	−0.58 (16)		

### Hydrogen-bond geometry (Å, °)

**Table:**

Cg1 is the centroid of the N1,N2/C7–C10 ring. Cg3 is the centroid of the C12–C17 ring.

**Table:**

*D*—H···*A*	*D*—H	H···*A*	*D*···*A*	*D*—H···*A*
N3—H1N3···N1^i^	0.909 (17)	2.147 (17)	3.0539 (14)	175.7 (14)
C5—H5A···Cg1^ii^	0.995 (14)	2.883 (15)	3.3595 (14)	110.3 (10)
C18—H18A···Cg3^iii^	0.960 (16)	2.846 (19)	3.7977 (16)	171.8 (13)

Symmetry codes: (i) −*x*+2, −*y*, −*z*; (ii) −*x*+2, −*y*, −*z*+1; (iii) *x*, *y*−1, *z*.
